# Transcriptomics driven identification of hub gene miRNA interactions for biomarker and therapeutic target discovery in gynecological cancers

**DOI:** 10.3389/fonc.2025.1719597

**Published:** 2026-01-05

**Authors:** Yuanjun Zhu, Sisi Chen, Mei Cao, Wangbo Liu, Hanling Huang, Ke Huang, Lingling Shi

**Affiliations:** 1 Department of Obstetrics and Gynecology, Taihe Hospital, Hubei University of Medicine, Shiyan, China; 2 Department of Emergency, Taihe Hospital, Hubei University of Medicine, Shiyan, China; 3 Department of Physical Examination Center, Taihe Hospital, Hubei University of Medicine, Shiyan, China; 4 Department of Ultrasound Medicine, Taihe Hospital, Hubei University of Medicine, Shiyan, China

**Keywords:** Cervical cancer, RNA sequence data, regression analysis, hub genes, miRNA, noncoding RNAs, potential biomarker, qRT-PCR

## Abstract

**Introduction:**

MicroRNAs (miRNAs) are small, single-stranded noncoding RNAs that play critical roles in disease development, including gynecological cancers like vulvar and cervical cancer. Their high heterogeneity makes achieving an accurate diagnosis difficult in modern clinical practice.

**Methods:**

In this study, we used *in silico* analyses to identify hub genes, miRNAs, and their interactions, enabling the discovery of potential biomarkers that may improve the diagnosis and treatment of cervical cancers following validation by quantitative gene expression analysis.

**Results:**

The statistical analysis of GEOR2 yielded 16,344 differentially expressed genes (DEGs), and through robust regression analysis, 229 common DEGs were retrieved. Among them, 94 and 135 genes were downregulated and upregulated, respectively. We retrieved ten hub genes via a protein–protein interaction network and cytohubba, namely CDK1, AURKA, BUB1B, CCNB1, TOP2A, KIF11, BUB1, CCNB2, CDCA8, and BIRC5. Following extensive *in silico* analysis, 30 miRNAs that interact with hub genes were identified and among these miRNAs, hsa-miR-653-5p, hsa-miR-495-3p, hsa-miR-381-3p, hsa-miR-1266-5p, and hsa-miR-589-3p were the top five interactive miRNAs that targeted the most hub genes and were involved in key functions leading to colorectal cancer, cervical cancer, glioma, and TGF-beta signaling. We further validated the differential expression of hub genes in HeLa and HeLaDP cells using real-time PCR (P < 0.01).

**Discussion:**

The identified miRNAs exhibit strong regulatory interactions with these hub genes, while serine/threonine protein kinases emerged as the most significantly associated group. Together, these findings highlight promising biomarker candidates and potential therapeutic targets for gynecological cancers.

## 1 Introduction

Cervical cancer (CC) is the fourth leading cause of cancer-related deaths among women worldwide,; approximately 483,000 new cases and 274,000 deaths have occurred in low- and middle-income countries in recent years, representing the highest burden globally (1, 2). Although global vaccination and screening programs have reduced the incidence of several gynecological cancers, CC remains a major public health challenge (2). The high-risk human papillomavirus is a sexually transmitted disease that primarily causes CC development. More than 200 genotypes have been identified, with approximatley 40 capable of infecting epithelial cells in the anogenital cells (3). Of the 40 genotypes 13 have been known as carcinogenic, and among them the HPV16 being the most common in squamous cell carcinoma (59.3%) and adenocarcinoma (36.3%) (4–6).

Clinical staging serves as a key indicator of the response to chemoradiotherapy (CRT) in CC, yet treatment resistance remains a significant challenge that impacts patient outcomes. The evidence shows that CRT effectiveness differs markedly among patients, even those with similar histological traits, leading to increased risks of metastasis and recurrence. This disparity contributes to higher mortality rates, particularly among women diagnosed late with advanced-stage CC, especially in low-income regions where access to treatment is limited. The variability in CRT success emphasizes the need for new biomarkers to guide personalized therapeutic strategies based on tumor characteristics and individual patient profiles (7, 8). MicroRNA genes constitute approximately 1% of the genomes of various animals and can target numerous conserved and non-conserved sequences, influencing approximately 30% of genes. Despite not coding for proteins, microRNAs play crucial roles in cellular function and may aid in cancer diagnosis, prognostication, and treatment target identification (9, 10). The interaction between microRNAs and messenger RNAs (mRNAs) is complex, with approximately 60% of mRNAs showing interactions with microRNAs, as confirmed by various studies. Additionally, multiple microRNAs may target the same gene, as indicated by analyses using computer algorithms (11, 12).

Therefore, the present study carried out a comparative analysis of three RNA sequence datasets associated with CC following DEGs analysis, protein–protein interaction to prediction of hub genes and their interaction and association with miRNA. Study revealed that not only hub genes but miRNAs such as hsa-miR-653-5p, hsa-miR-495-3p, hsa-miR-381-3p, hsa-miR- 1266-5p, hsa-miR-589-3p top five upregulated and hsa-miR-1-5p, has-miR-300, hsa-miR-3913-3p, hsa-miR-6871-5p, hsa-miR-3922-5p top five downregulated could be a potential therapeutic targets and biomarkers in gynecological cancers.

## 2 Materials and methods

**Figure 1:**
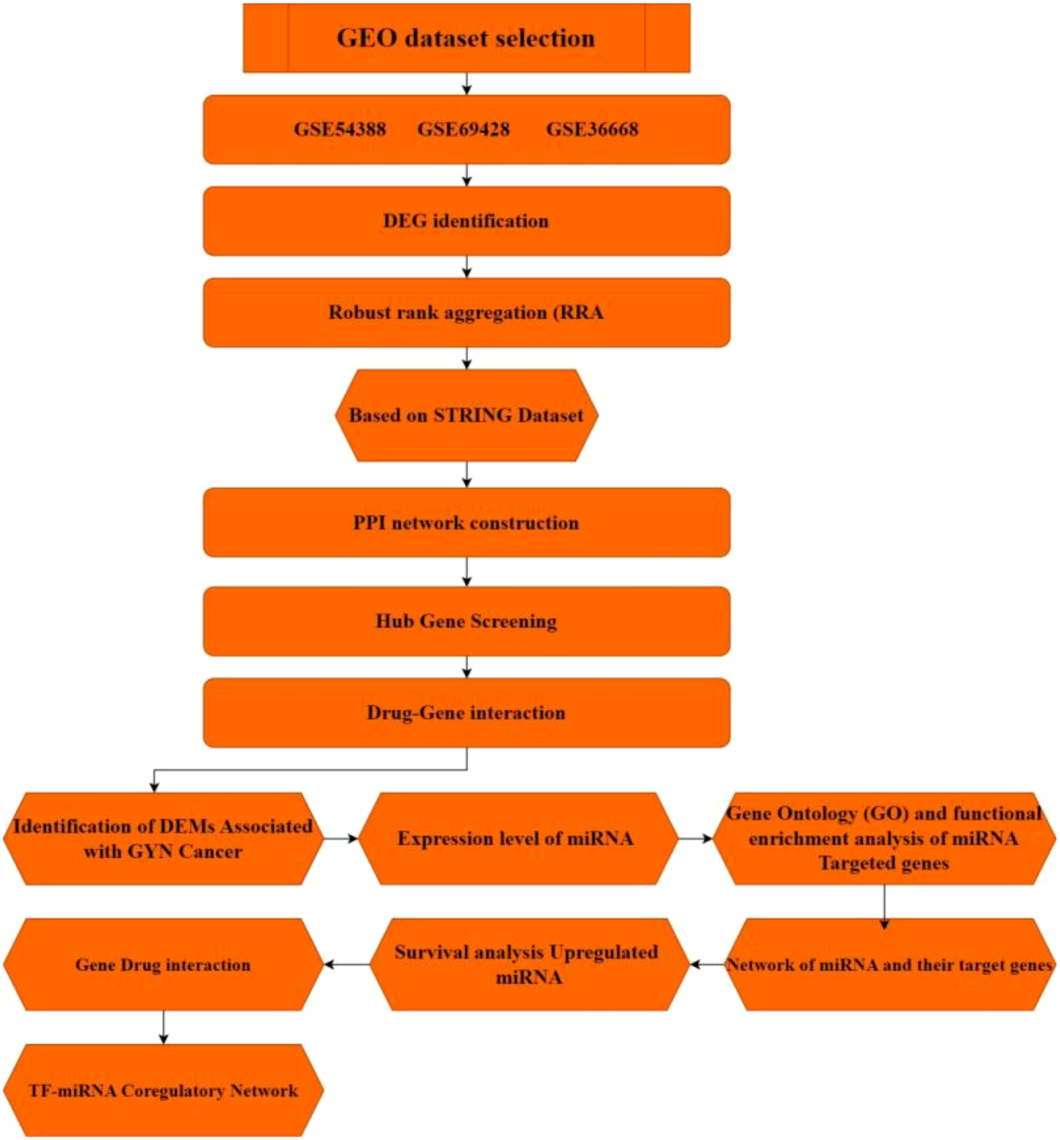
Representation of the workflow of the whole process used to achieve study objectives.

The schematic representation of the entire project is described in **Figure 1**.

### 2.1 DEG identification

Three gene expression profile datasets for GNY (GSE54388, GSE69428, and GSE36668) were obtained from the well-reputed database Gene Expression Omnibus (GEO), which has been reported for various cervical-associated diseases. The distinct selection criteria were as follows: (1) control, normal GYN test, and GYN-infected samples; (2) type of test used (expression profiling by array); and (3) “*Homo sapiens*” as species selection. The notes of the chip platform were GPL 570 and GPL 570, Pearl (ver. 5. 30). The chip where the microarray assay was performed was used to collect the gene symbol by translating the microarray probe name, and DEGs were identified with the program limma from the R package after differential expression. The selection standard was |log2 (FC)| > 1, where the p-value was <0.05. The cut off of |log_2_(FC)| > 1 corresponds to a minimum two-fold change in expression, which is widely considered a stringent criterion of substantial transcriptional DEGs rather than minor variations or noise inherent in microarray data (13). The p-value < 0.05 criterion is used to retain only statistically reliable genes whose differential expression is unlikely due to random variation (14).

### 2.2 Robustness analysis via rank aggregation

Additionally, they filtered out common DEGs between two datasets via a method called robust rank aggregation (RRA). A probability model is used by the RRA method, which is the result of different ranking lists (15). Some studies reported positive outcomes after researchers worked on genetic lists from the array of chip data. Therefore, this report used the RRA approach to detect DEGs that were more relevant to the two microarray datasets. Using the R package RRA, genes were sequenced and pronounced robust DEGs whose genes were sequenced in the two datasets were identified. Next, the upregulated and downregulated genes from each dataset were systematically ranked on the basis of the FC ranking. The genes with a | log2 (FC) |>1 and a p value <0.05 were considered to be the most significantly differentially expressed genes (DEGs) (16).

### 2.3 Module analysis of the protein–protein interaction network

The modules associated with the common DEGs from the three datasets were analyzed to identify key biological processes, pathways, and molecular functions. Identifying such clusters provides insights into the underlying molecular mechanisms and highlights potential targets for further investigation. The robust DEGs were uploaded to the STRING online tool, with medium confidence (0.400) and a minimum required interaction score, to construct a protein–protein interaction (PPI) network, enabling the visualization of potential functional associations among the encoded proteins (17). The analysis revealed that no additional network nodes were available for peers within the PPI network, indicating that the uploaded DEGs represented a distinct and specific interaction cluster. The term *“Homo sapiens”* was selected, and the confidence threshold was set to “highest confidence.” The relevant PPI network data column was then extracted, saved as a TSV file, and visualized using Cytoscape (v3.8) (18). After the TSV data files were imported into Cytoscape, cluster modules, which are the main aspects of the network were constructed via the MCODE plugin (19). To identify the genes associated with the core network modules, the modules were first annotated by the KEGG signaling pathway and further analyzed by the R programming language. Differences with a p value <0.05 were considered statistically significant (20).

### 2.4 Screening of hub genes and analysis

Hub gene screening was conducted to identify key genes with high connectivity and central roles within the PPI network that are likely critical regulators involved in biological processes related to gynecological cancer, making them potential targets for further functional and therapeutic analysis (21). The Cytoscape plug-in CytoHubba offers several topological analysis algorithms, such as Euclidean Percolated Component (EPC), Density of Maximum Neighborhood Component (DMNC), Degree, Maximum Neighborhood Component (MNC) and Maximal Clique Centrality (MCC), and six centralities: Betweenness, Stress, Closeness, Radiality, BotcceNeck and ECCentricity (22). Molecular complex detection (MCODE) (degree cutoff = 2, max depth = 100, node score cutoff = 0.2 and K-core = 2) was applied.

### 2.5 Drug-hub gene interactions

The identified hub genes were considered potential targets for drug discovery via the DGIdb database (23). This database contains drug-gene interaction data from 30 diverse sources, including ChEMBL, DrugBank, Ensembl, NCBI Entrez, PharmGKB, and NCBI PubMed. Medications corroborated by at least two databases or PubMed references were validated as candidate potential drug. The final list exclusively comprised medications approved by the Food and Drug Administration. The identified target gene network was developed using the STITCH database (24), which also integrates drug–gene connections.

### 2.6 Prediction of miRNAs, functional annotations, and analysis

The prediction of miRNAs targeting the identified hub genes was carried out to explore potential regulatory interactions involved in posttranscriptional gene regulation. Considering the list of hub genes, we utilized the miRDB database to predict the miRNAs associated with the hub genes. miRDB is an essential resource for functional annotation or target prediction. It contains predicted miRNA targets for five species: humans, mice, rats, dogs, and chickens (25). miRDB provides hundreds of cell line expression profiles, and targeted miRNAs can be identified in desired cell lines by setting appropriate search criteria (26). We carried out our extensive analysis over and targeted 12,000 genes to identify associations with hub genes following the identification of target genes associated with miRNAs. The predicted miRNAs associated with the hub genes were further analyzed for functional characterization via DIANA-miRPath v3.0, which enables pathway enrichment analysis and functional annotation. This tool enables the identification of key biological processes and signaling pathways regulated by miRNAs, shedding light on their potential contributions to the molecular mechanisms fundamental to GNYn (25). Moreover, the tool has significantly extended support to comprehensive analysis of KEGG molecular pathways and multiple aspects of Gene Ontology (GO) in seven species (*Drosophila melanogaster, Caenorhabditis elegans, Mus musculus, Rattus norvegicus, Homo sapiens, Gallus gallus* and *Danio rerio*).

### 2.7 Prediction and analysis of differentially expressed miRNAs and their survival analysis

The functionally enriched miRNAs were analyzed via the database of differentially expressed miRNAs in human cancers (dbDEMC) (27). It is a comprehensive resource designed to store and display differentially expressed miRNAs in cancers, identified by high-throughput and low-throughput sequencing technologies. In this study, we applied and explored 403 miRNA expression datasets retrieved from public repositories, including ArrayExpress, The Cancer Genome Atlas (TCGA), Sequence Read Archive (SRA), and GEO (28). Moreover, the easy-to-use and simple web application ExplORRNet offers an in-depth analysis of dysregulated miRNA functional associations and survival in patients with breast and gynecological malignancies (29). In addition to being useful for fundamental and clinical data, the ExplorRNet tool has helped the cancer genomics community as a whole by facilitating, promoting, and mining fast-expanding public datasets to advance the science of precision oncology. The final predicted differentially expressed upregulated miRNAs were utilized for survival analysis.

### 2.8 Validation of hub genes by quantitative real time PCR analysis

Immortalized cancer cell lines have been instrumental in cervical cancer research and drug discovery. The most commonly used human cell line in cancer research, HeLa, was obtained from Servicebio (Hubei Wuhan), and experiments were performed as described by Zhang et al. (30). All the cell lines were cultured in DMEM (HyClone, Logan, U.S.) supplemented with 10% fetal bovine serum (FBS, Gibco), 100 U/mL penicillin, and 100 U/mL streptomycin (Invitrogen, U.S.), after which they were incubated at 5% CO^2^ and 37 °C with saturated humidity. All experiments were performed with mycoplasma-free cells. Total RNA from the HeLa and HeLa-DPcell lines was extracted via the RNA Quick Purification Kit (ES Science, Shanghai, China), and cDNA was synthesized via the cDNA Reverse Transcription Kit (Vazyme, Nanjing, China). RT–qPCR was performed via TB Green™ Premix Ex Taq™ II (RR420A; Takara, China) with specific primers (**Table 1**) on a Bio-Rad CFX96 Real-time PCR system (Bio-Rad, USA) following the manufacturer’s instructions.

**Table 1:** List of hub gene primers used for quantitative real time PCR analysis.

No.	GENE	SEQUENCE
1	H-GAPDH-F	TGTTGCCATCAATGACCCCTT
	H-GAPDH-R	CTCCACGACGTACTCAGCG
2	H-CDK1-F	GGAAACCAGGAAGCCTAGCATC
	H-CDK1-R	GGATGATTCAGTGCCATTTTGCC
3	H-AURKA-F	AGTTGGAGGTCCAAAACGTG
	H-AURKA-R	ATTCTGAACCGGCTTGTGAC
4	H-BUB1B-F	GTGGAAGAGACTGCACAACAGC
	H-BUB1B-R	TCAGACGCTTGCTGATGGCTCT
5	H-CCNB1-F	GACCTGTGTCAGGCTTTCTCTG
	H-CCNB1-R	GGTATTTTGGTCTGACTGCTTGC
6	H-TOPA-F	GAGGTCCCAAAGATAAGCCAGG
	H-TOPA-R	CTGGCTTTATCCATCCACAGTCC
7	H-KIF11-F	TACAGAAACCACTTAGTAGTGTCC
	H-KIF11-R	GAGTTCCTGTGAGAAGCCATCAG
8	H-BUB1-F	GCTCTGTCAGCAGACTTCCTTC
	H-BUB1-R	CAGCAGATGTGAAGTCTCCTGG
9	H-CCNB2-F	CAACCAGAGCAGCACAAGTAGC
	H-CCNB2-R	GGAGCCAACTTTTCCATCTGTAC
10	H-CDCA8-F	CAGTGACTTGCAGAGGCACAGT
	H-CDCA8-R	CTCATTTGTGGGTCCGTATGCTG
11	H-BIRC5-F	GATGACGACCCCATGCAAAG
	H-BIRC5-R	CGCACTTTCTCCGCAGTTTC

The Ct values of the identified hub genes were normalized to those of GAPDH, an internal control gene, and data were analyzed via the 2−ΔΔCT method. The names of the genes used in this study are CDK1, AURKA, BUB1B, CCNB1, TOP2A, KIF11, BUB1, CCNB2, CDCA8, and BIRC5.

## 3 Results and discussion

### 3.1 Data pre-processing and DEG identification

With the advancement of high-throughput sequencing technologies such as RNA sequencing and microarrays in combination with bioinformatics analysis, many datasets have been generated, including lncRNA, miRNA, and mRNA expression profiles. Millions of genes have been detected and are widely used to predict potential biomarkers for various types of cancer (31). For the identification of the hub genes involved in gynecological cancer, the GEO datasets GSE54388, GSE69428, and GSE36668 were utilized. This retrieved dataset included different samples, including control samples and gynecological carcinoma samples, and was subjected to statistical analysis via GEO2R. All DEG IDs, gene log fold change (FC) values, and p values.

**Figure 2:**
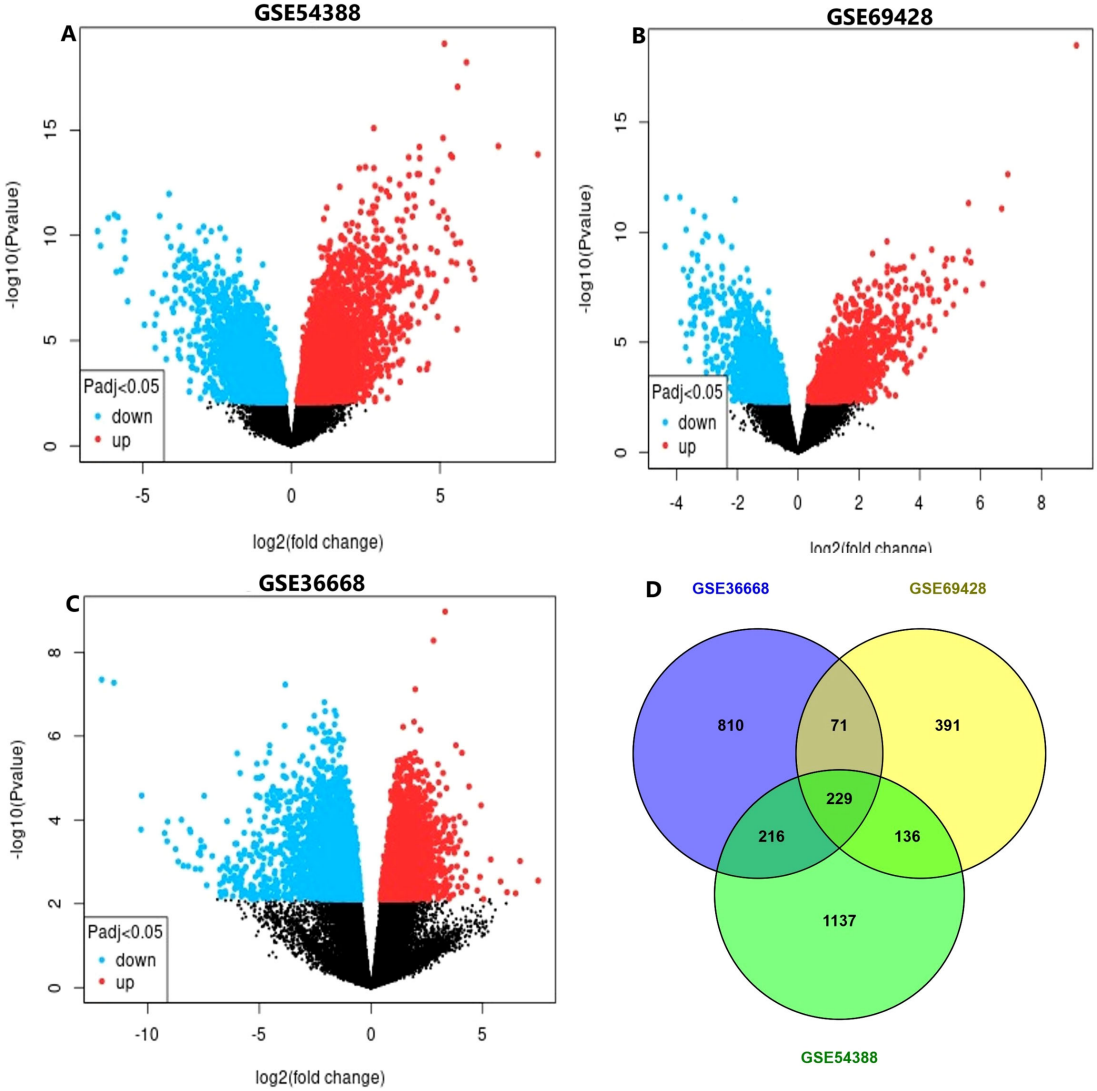
Differential expression analysis and identification of shared DEGs across datasets. **(A–C)** Volcano plots showing DEGs in the GSE54388, GSE69428, and GSE36668 datasets, respectively. Each plot displays the log 2 fold change on the x-axis and the –log10 (p-value) on the y-axis. Significantly upregulated genes (Padj < 0.05) are shown in red, significantly downregulated genes in blue, and non-significant genes are shown in black. **(D)** Venn diagram illustrating the overlap of DEGs among the three datasets. A total of 229 genes were commonly dysregulated across all datasets, with additional unique and partially shared DEGs identified in each dataset.

The gene volcano plot shown in **Figures 2A–C** shown in red and green indicating increased and decreased gene expression, respectively, whereas black indicates genes whose expression did not change. This analysis provides a foundational step for identifying potential hub genes involved in gynecological cancers, offering insights into possible biomarkers or therapeutic targets. The statistical analysis yielded 16344 DEGs from the three designated studies by setting a cut-off p value <0.05 and their distribution is described in **Figure 2D**.

### 3.2 Robust rank aggregation

Through the RRA, 229 common DEGs were identified in the datasets for GYN compared with those for the tissue samples (**Figure 2D**). The list contains not only indispensable genes such as transporters, energy-associated genes, and transcription factors but also several key genes that are known to be associated with various types of cancers, such as decorin, mimecan, and serine/threonine protein kinases. Decorin is an extracellular matrix small leucine-rich proteoglycan protein. It affects several types of cancer by targeting signaling molecules involved in angiogenesis, metastasis, survival, and cell growth (32). Similarly, mimecan is a significant precursor lesion of colorectal cancer, which is among the most common cancers worldwide. PIM kinases, which belong to the serine/threonine kinase family, are overexpressed in several cancers, including prostate, breast, colon, endometrial, and gastric cancers (33, 34). The identification of potential differentially expressed key genes associated with various types of cancer reveals the value of the dataset chosen.

### 3.3 Module analysis and PPI network construction

**Figure 3:**
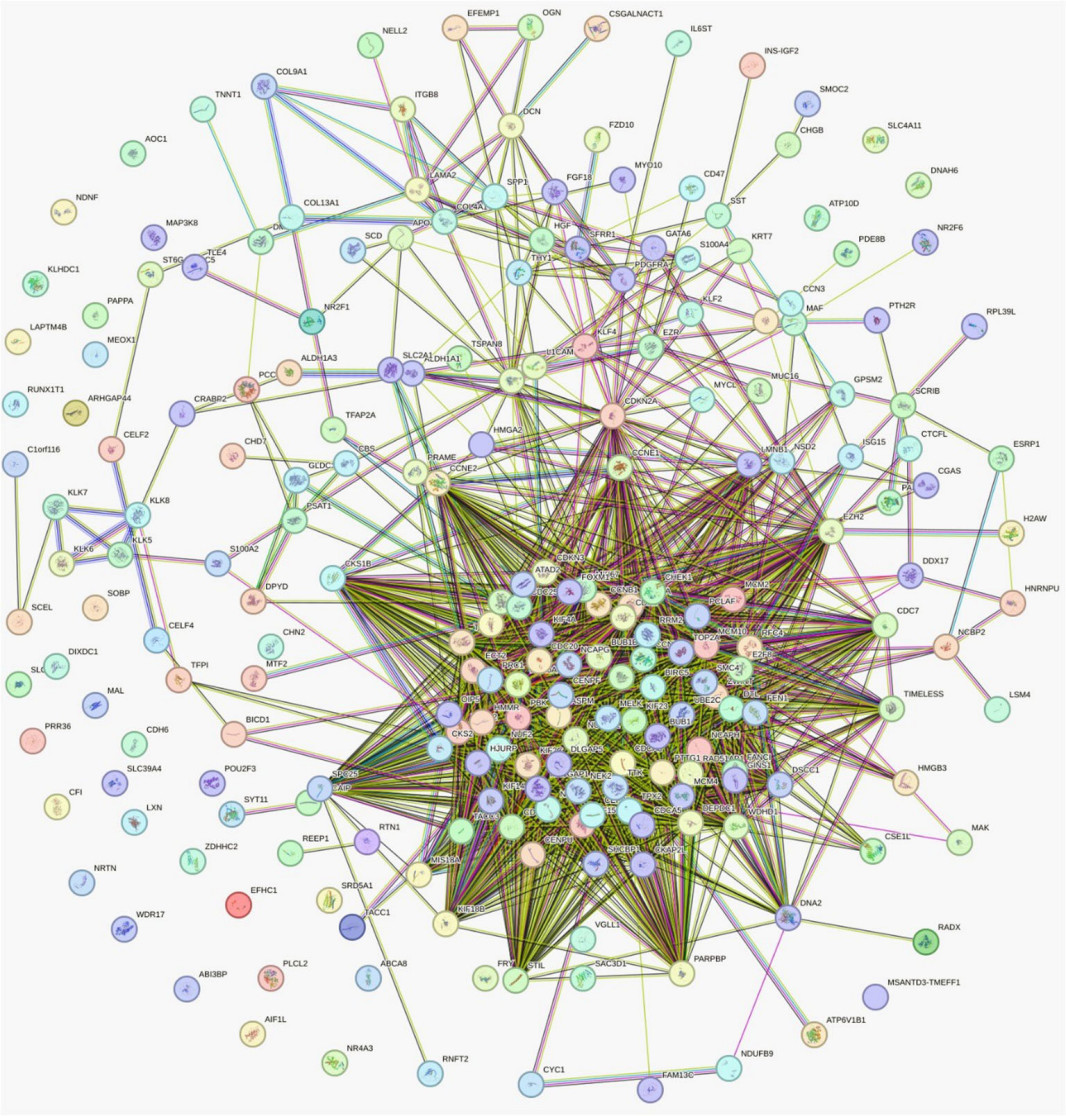
The PPI network representing the interactions among the common DEGs identified across the included datasets. Nodes represent proteins encoded by DEGs, and edges indicate known or predicted interactions on the basis of experimental, database, coexpression, neighborhood, and text-mining evidence. The densely connected core indicates highly interactive proteins that may serve as functional hubs in the disease network, whereas peripheral nodes represent less connected but potentially important regulators. This network highlights key molecular clusters and potential hub targets involved in the biological processes underlying the studied conditions.

The PPI network constructed from the STRING database using the DEGs to assess interactions and conduct a comprehensive analysis is illustrated in **Figure 3**. The Cytoscape ClusterViz module and MCODE algorithm were used to visualize robust DEGs, resulting in a TSV list that was imported into the software. The average local clustering coefficient was 0.563, reflecting a moderate tendency for nodes to cluster together. With the expected number of edges being 511, the observed count of 3014 edges significantly surpasses this, suggesting an exceptionally dense network. Furthermore, a *p* value of PPI enrichment less than 1.0^e-16^ indicated highly substantial PPI enrichment within the network. To reliably categorize and diagnose diseases via molecular data and provide insight into the mechanisms of disease pathogenesis, identifying network biomarkers is imperative. The results of the PPI enrichment analysis revealed a persistent correlation between various genes and gene function.

Overall, key information noted from the network such as the dense region in the center with genes BUB1, KIF11 etc., suggests the presence of hub proteins or a central complex involving many interactions. Peripheral nodes with fewer connections might represent specialized proteins that interact with the core network indirectly and the diverse colors and arrangements suggest that this visualization emphasizes different functional groups or sub-pathways.

### 3.4 Prediction and analysis of the hub genes

**Figure 4:**
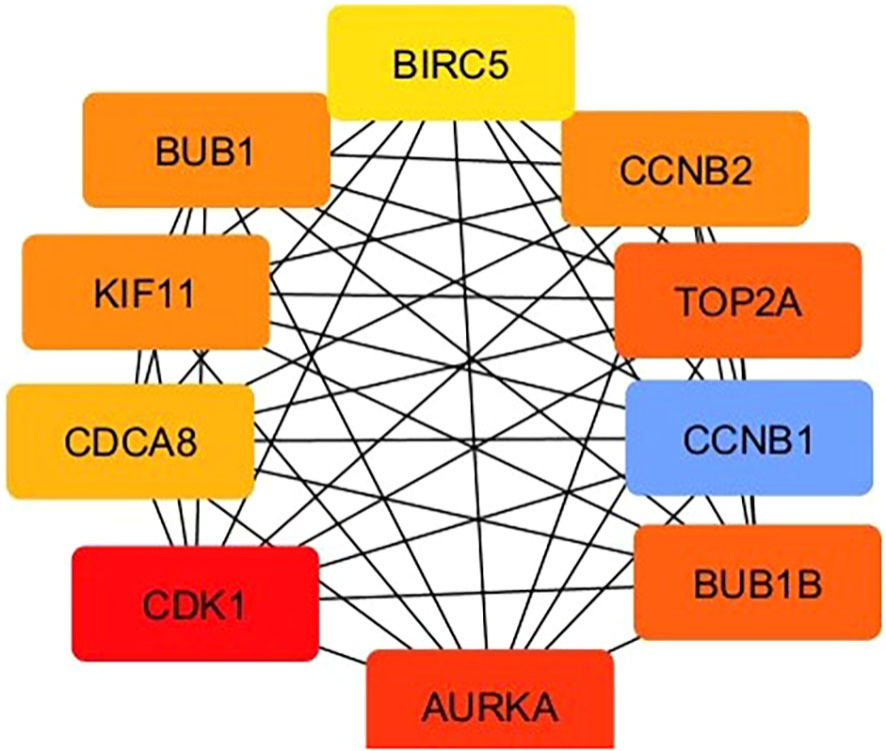
Identification of Hub genes. Hub genes were identified by the intersection of 229 genes from 10 algorithms including MCC, DMNC, MNC, degree, EPC, botcceNeck, eccentricity, closeness, radiality, and betweenness.

With a set of topological analysis algorithms that are part of the Cytoscape plug-in, CytoHubba, which is used for the identification of significant nodes in PPI networks, assigns a score to each node belonging to the PPI network and is considered a hub gene. The results revealed 10 upregulated hub genes, namely, TOP2A, BIRC5, KIF11, CDK1, AURKA, BUB1B, CCNB1, BUB1, CCNB2 and CDCA8 which are shown in **Figure 4**.

Cyclin-dependent kinases (CDKs), aurora kinases, BUB1B A, CCNB1, BUB1, CCNB2, and CDCA8 are serine/threonine kinases that are considered promising targets for cancer therapy. These proteins are essential for cell cycle progression, particularly when they form complexes with cyclins (35, 36). The identification of hub genes provides valuable insights into the molecular mechanisms underlying cervical and gynecological cancers. These genes play critical roles in various biological processes, especially those involved in cell cycle regulation, mitotic progression, and apoptosis. For example, TOP2A not only plays a pivotal role in cell proliferation, and recombination but also plays a role in chromosome separation and concentration and is known to play an oncogenic role in several tumor types (37). Survivin is another name for baculoviral inhibitor of apoptosis repeat containing 5 (BIRC5) and is known to play a role in preventing caspase activation and adversely controlling programmed cell death or apoptosis. Owing to these characteristics, BIRC5 overexpression promotes particular division and survival pathways linked to cancerous tumors (38). KIF11 is one of the 45 kinesin superfamily proteins that functions as a motor protein in mitosis and emerging evidence has revealed that KIF11 plays pivotal roles in cancer initiation, development, and progression. BIRC5 and AURKA play roles in addition to their roles in tumor cells, influencing the immune response and angiogenesis (39, 40). This opens avenues for combining these targets with immunotherapy or antiangiogenic agents.

Among the other 10 hub genes, cyclin-dependent kinase (CDK1), aurora kinase (AURK), BUB1, BUB1B, CCNB1, CDCA8, CDK1, and CCNB2 are a family of serin-threonin kinases and key regulators of mitotic spindle formation. The overexpression of these genes is frequently associated with prognosis in various tumors (41) and with adverse clinical outcomes in various solid tumors (42). For example, CDK1 is highly expressed in various cancers, including Hodgkin’s lymphoma, colorectal cancer, prostate cancer, gastric lymphoma, childhood acute lymphoblastic leukemia, and epithelial ovarian cancer (43). The literature shows that these genes may help to overcome drug resistance and improve treatment outcomes, drug-gene interactions and cytotoxic effects, and understanding these genes can lead to the discovery of novel therapeutic targets and strategies.

### 3.5 Gene-Drug interaction and network analysis

**Figure 5:**
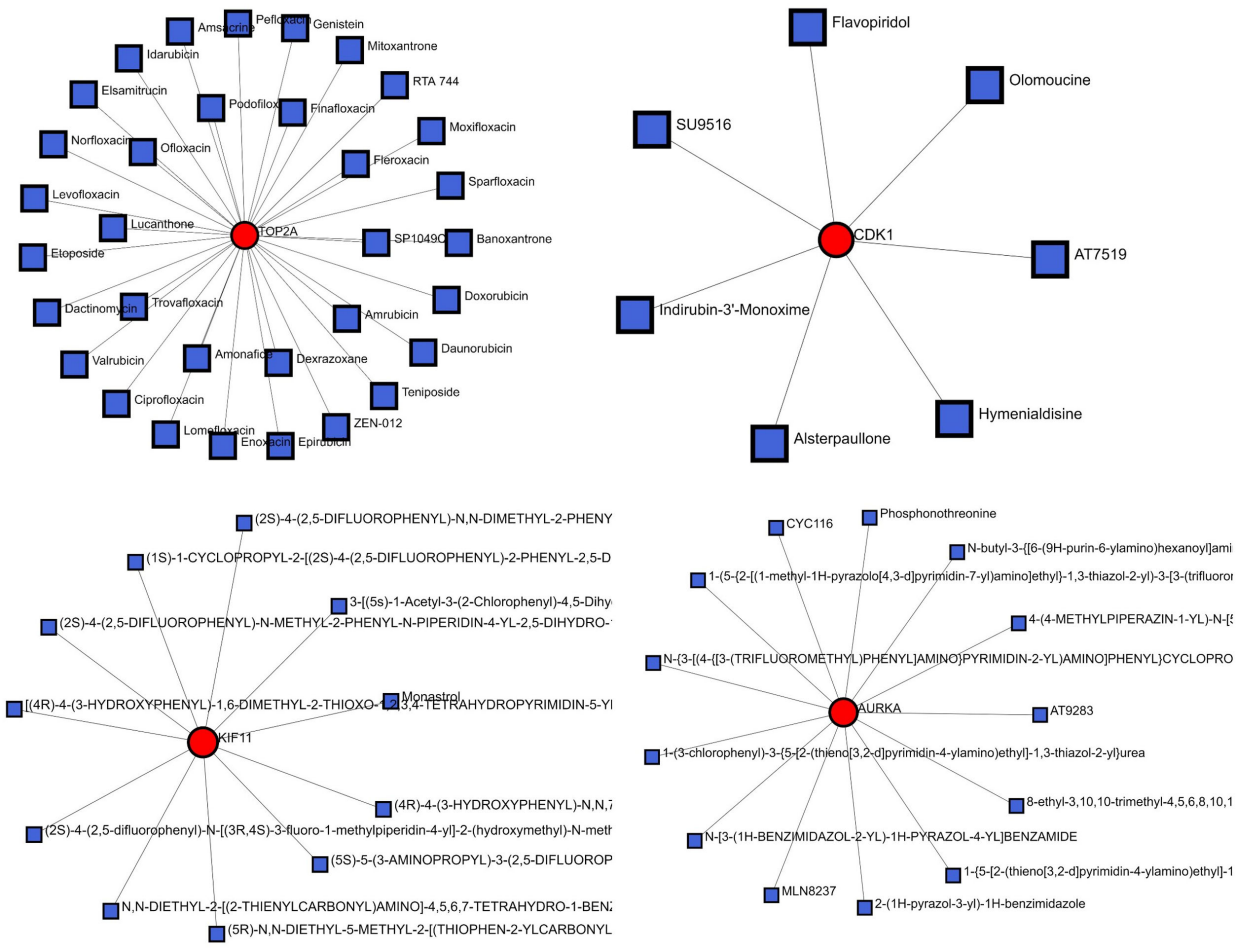
Gene-drug interaction network-specific genes that interact with various drugs.

Gene-Drug interaction networks are generated via tools such as NetworkAnalyst. It visualizes how specific genes interact with various drugs. The highlighted genes (TOP2A, AURKA, CDK1, and KIF11) are shown in yellow, indicating their central role in the network, while the drugs that interact with these genes are displayed in red. For example, TOP2A interacts with a variety of chemotherapeutic agents, such as etoposide, doxorubicin, and levofloxacin, all of which interfere with DNA replication in cancer cells (44). AURKA is associated with drugs such as MLN8237 and phosphorothioate, both of which are used in cancer treatment because of their impact on cell division (45). CDK1, another key player, interacts with inhibitors such as flavopiridol and AT7519, which have been studied for their potential in cancer therapies that target cell cycle regulation (46). KIF11 interacts with drugs, including monastrol and dimethylenastron, which inhibit kinesin family members involved in mitosis. These networks highlight critical drug-gene interactions, emphasizing their importance in developing targeted cancer therapies as shown in **Figure 5**.

Moreover, the cytotoxic effects of potential inhibitors of these hub genes were also investigated in this study, and CDK1, BUB1 and TOP2A seem to be potential cytotoxic effects. For example, the inhibition of CDC25, an activator of CDK1, leads to a reduction in the growth of pancreatic cancer cell lines, indicating the potential of CDK1 inhibition as a novel drug target to treat cancers (47). However, there is only scarce clinical evidence for the concrete benefits of CDK1 inhibition, which is a cytotoxic effect of CDK1 inhibitors on healthy cells (Wijnin). Similarly, an inhibitor of BUB1 increases the cytotoxic ability of different classes of chemotherapy, targeted agents, and radiotherapy in triple-negative breast cancer, which is the most difficult subtype of breast cancer to treat owing to its lack of expression of targetable genes (48). There are reports regarding TOP2, which is a drug target for many types of cancers. However, clinically used TOP2 inhibitors not only kill cancer cells, but also damage normal cells, and can even give rise to other types of cancers (49).

Overall, we believe that the inhibitors associated with the AURKA, CCNB1, CCNB2, KIF11, CDCA8, and BIRC5 genes may be potential targets for future studies to treat cancer. However, there is limited research that thoroughly explains the molecular mechanisms of CDK1, TOP2A, BUB1, and BUB1B inhibitors and in depth studies in combination with computer aided drug desgine can further explore and optimize the potential ability of these inhibitors for gynecological cancers, as they can strongly inhibit cancer cell growth but have low side effects.

Biomarker guided approaches can significantly accelerate early phase clinical testing by enabling the rapid identification of patients who are most likely to benefit from a given therapy. The selection of individuals whose tumors express specific molecular signatures such as gene mutations, dysregulated miRNAs, or circulating markers can reduce heterogeneity in study cohorts, thereby increasing the probability of detecting early signals of efficacy (50, 51). Moreover, biomarker-based stratification allows patients to be grouped according to biological risk or therapeutic sensitivity, improving the precision of outcome interpretation and enabling more personalized treatment strategies (52, 53). Together, these advantages streamline early-phase development while setting the foundation for more effective, individualized clinical care.

### 3.6 Identification of differentially expressed miRNAs and their functionalities

For the identification of miRNAs involved in gynecological cancer and their associations with various hub genes, the same GEO datasets, GSE54388, GSE69428, and GSE36668 were utilized. A total of 30 miRNAs were associated with GSE54388, GSE69428, and GSE36668 and linked to ten hub genes, which were further classified into downregulated and upregulated miRNAs on the basis of the log Fc and **p** values (**Table 2**). The expression levels of these 30 upregulated and downregulated miRNAs are shown in **Table 2**.

**Table 2:** Represent the top 30 miRNAs associated with hub genes and with their evaluation score.

Target rank	Target score	miRNA	Gene symbol	Gene description
1	96	hsa-miR-5011-5p	CDK1	cyclin-dependent kinase 1
2	95	hsa-miR-3913-3p	CDK1	cyclin-dependent kinase 1
3	94	hsa-miR-4760-3p	CDK1	cyclin-dependent kinase 1
4	97	hsa-miR-3941	AURKA	aurora kinase A
5	95	hsa-miR-6871-5p	AURKA	aurora kinase A
6	91	hsa-miR-1-5p	AURKA	aurora kinase A
7	87	hsa-miR-524-5p	BUB1B	Serine/threonine kinase B
8	87	hsa-miR-520d-5p	BUB1B	Serine/threonine kinase B
9	85	hsa-miR-3922-5p	BUB1B	Serine/threonine kinase B
10	98	hsa-miR-548n	CCNB1	cyclin B1
11	96	hsa-miR-559	CCNB1	cyclin B1
12	95	hsa-miR-548ar-5p	CCNB1	cyclin B1
13	92	hsa-miR-8077	TOP2A	DNA topoisomerase II alpha
14	91	hsa-miR-4641	TOP2A	DNA topoisomerase II alpha
15	89	hsa-miR-4663	TOP2A	DNA topoisomerase II alpha
16	98	hsa-miR-190a-3p	KIF11	kinesin family member 11
17	97	hsa-miR-381-3p	KIF11	kinesin family member 11
18	97	hsa-miR-300	KIF11	kinesin family member 11
19	94	hsa-miR-5688	BUB1	BUB1 mitotic checkpoint serine/threonine kinase
20	93	hsa-miR-495-3p	BUB1	BUB1 mitotic checkpoint serine/threonine kinase
21	92	hsa-miR-653-5p	BUB1	BUB1 mitotic checkpoint serine/threonine kinase
22	97	hsa-miR-670-3p	CCNB2	cyclin B2
23	91	hsa-miR-4251	CCNB2	cyclin B2
24	72	hsa-miR-10525-3p	CCNB2	cyclin B2
25	92	hsa-miR-4518	CDCA8	cell division cycle associated 8
26	92	hsa-miR-1266-5p	CDCA8	cell division cycle associated with 8
27	91	hsa-miR-589-3p	CDCA8	cell division cycle associated with 8
28	93	hsa-miR-548t-3p	BIRC5	baculoviral IAP repeat containing 5
29	93	hsa-miR-548ap-3p	BIRC5	baculoviral IAP repeat containing 5
30	93	hsa-miR-548aa	BIRC5	baculoviral IAP repeat containing 5

miRNAs with more than 80% target scores, such as hsa-miR-190a-3p, had a target score of 98, hsa-miR-670-3p had a target score of 97, and the minimum target score was hsa-miR-3922-5p 85. All the miRNAs were more or less associated with the hub genes CDK1, AURKA, BUB1B, CCNB1, TOP2A, KIF11, BUB1, CCNB2, CDCA8, and BIRC5. The most important element to be noted was serine/threonine protein kinase, which is associated with most hub genes, followed by those linked with miRNAs. The interactions between microRNAs (miRNAs) and genes in a network of 379 nodes, 420 edges, and 10 seed nodes were analyzed. This study reveals the efficacy of miRNA-gene interactions and the functions of genes, providing crucial insights for understanding regulatory networks involving miRNAs in gene expression and cellular processes. Consequently, miRNAs are invaluable resources for researchers exploring the impacts of miRNAs on biological functions and disease mechanisms, as described in **Table 2**.

The GO functional enrichment of the top upregulated and downregulated differentially expressed miRNAs was analyzed via the DIANA-mirPathV3 database. The enrichment of biological processes for five highly enriched upregulated miRNAs included process such as the Fc-gamma receptor signaling pathway for hsa-miR-653-5p, positive regulation of transcription, DNA-templated for hsa-miR-381-3p, and the toll-like receptor signaling pathway for hsa-miR-495-3p. The enrichment of the cellular component “organelle” in hsa-miR-653-5p, the protein complex in hsa-miR-589-3p, and the nucleoplasm in hsa-miR-381-3p. The enriched molecular functions included nucleic acid binding transcription factor activity in hsa-miR-653-5p, protein binding transcription factor activity in hsa-miR-381-3p, and RNA binding in hsa-miR-495-3p. MicroRNAs such as hsa-miR-653-5p or hsa-miR-495-3p are increasingly recognized for their dual relevance as both intratumoral regulators and circulating biomarkers. Emerging studies have demonstrated that miR-653-5p is dysregulated in multiple tumor types, where it promotes proliferation, metastasis, and therapy resistance (54, 55). Importantly, miRNAs aberrantly expressed in tumors can also be released into the bloodstream via exosomes or apoptotic bodies, where they remain stable and readily detectable in circulating fractions. Blood-based diagnostics, particularly liquid biopsies, offer a minimally invasive approach for detecting and monitoring neuroblastoma (56, 57).Tumor–stroma interactions add another layer, as crosstalk with fibroblasts, immune, and endothelial cells can also influence the release and profile of circulating biomarkers (58). By analyzing circulating tumor RNA, and tumor cells present in peripheral blood, liquid biopsies can capture key genetic alterations reflective of the tumor. MicroRNAs such as hsa-miR-653-5p or hsa-miR-495-3p could provide valuable real-time information for diagnosis, treatment selection, and assessment of minimal residual disease, relapse, and recurrence (59).

The biological processes associated with the downregulated miRNAs are related to transcription, the response to stress, signaling pathways, and catabolic processes, indicating strong associations of these miRNAs with these functions. The cellular components with the highest enrichment were organelles, protein complexes, and nucleoplasm, suggesting strong associations of these miRNAs with these cellular structures. The molecular functions with the highest enrichment included nucleic acid binding transcription factor activity, protein binding transcription factor activity, and ion binding, highlighting the strong associations of these miRNAs with these molecular activities. The KEGG pathway of the upregulated miRNAs highlights the regulation of critical pathways i.e. key processes such as cell cycle regulation, apoptosis, or specific cancer-related signaling pathways, while downregulated miRNAs emphasize their regulatory role in gene expression and cellular function.

Previous studies revealed that each miRNA can regulate several hundred gene targets and participate in various gene signaling pathways (60). Consequently, miRNAs influence numerous biological functions, including apoptosis, differentiation, and cell proliferation (61). Altered miRNA expression can significantly impact cellular functional activity. Genomic research on human cancer has shown variability in miRNA expression (62). Extensive diagnostic and prognostic biological information are linked to miRNA expression. Thus, miRNA expression could serve as a predictor of cervical cancer prognosis, as imbalances in miRNA expression are common across all tumor types (63). As a result, these expression characteristics may be useful for potential value in diagnosis and prognosis.

### 3.7 Analysis of upregulated and downregulated miRNAs

The dbDEMC database identified top five up and downregulated genes in cancers, and using Cytoscape, a regulatory network was built for both upregulated and downregulated miRNAs. The results revealed the top five upregulated miRNAs (hsa-miR-653-5p, hsa-miR-495-3p,hsa-miR-381-3p, hsa-miR-1266-5p, hsa-miR-589-3p) regulate approximately 229 common genes including hub genes in the network, displaying interconnections between the genes and miRNAs. While the top five (hsa-miR-1-5p, hsa-miR-300, hsa-miR-3913-3p, hsa-miR-6871-5p, hsa-miR-3922-5p) downregulated miRNAs have no proper interconnection. These upregulated miRNAs show potential as biomarkers and therapeutic targets in gynecological cancers as shown in **Table 3**.

**Table 3:** Expression level of ten shortlisted miRNAs and their IDs.

miRNA ID	Source ID	Cancer subtype	Design	LogFC	Expression status	Expression ID
hsa-miR-653-5p	GSE106817	GYN/ovarian cancer	Blood	0.76	Up	EXP00527
hsa-miR-495-3p	GSE106817	GYN/ovarian cancer	Blood	0.6	Up	EXP00528
hsa-miR-381-3p	GSE106817	GYN/ovarian cancer	Blood	1.05	Up	EXP00528
hsa-miR-1266-5p	GSE31801	GYN/ovarian cancer	Blood	0.05	Up	EXP00327
hsa-miR-589-3p	GSE113486	GYN/ovarian cancer	Blood	1.38	Up	EXP00537
hsa-miR-1-5p	DRP001085	Bladder cancer	Cancer vs normal	0	Down	EXP00706
hsa-miR-300	GSE5244	Uterus cancer	cancer vs normal	-0.46	Down	EXP00030
hsa-miR-3913-3p	GSE106817	GYN/Ovarian cancer	Blood	-0.91	Down	EXP00528
hsa-miR-6871-5p	GSE106817	Ovarian cancer	Blood	-1.85	Down	EXP00528
hsa-miR-3922-5p	GSE113486	GYN/Ovarian cancer	Blood	-1.49	Down	EXP00537

Studies revealed the emerging role of hsa-miR-653-5p, hsa-miR-495-3p, and hsa-miR-381-3p in various cancer diseases. Such as hsa-miR-653-5p has been connected to aggressive tumors in human cancer and at the same time, it has an inhibitory effect on some other types of cancer, such as lung cancers, renal, liver, breast, and cervical (64) which is contrary to our study where upregulation of miR-653 showed aggressiveness in cervical cancer. The hsa-miR-495-3p is known to have inhibitory effects in the progression of various types of cancer such as colorectal cancer (65, 66), while we noted that it is upregulated in our findings. In the case of miR-381-3p it is also known to inhibit breast cancer progression (65) while in our study it is upregulated. Contrary to above mentioned miRNAs, literature verified that the functional effects of miR-1266, such as enhanced invasiveness and cell mobility, underscore its potential as a biomarker for aggressive cervical cancer. Moreover, its targeting to disabled homolog 2-interacting protein (DAB2IP) suggests a therapeutic avenue where inhibitors of miR-1266 or restoration of DAB2IP activity could suppress tumor progression (67). As for as miR-589-3p is cocnern, several studies have highlighted the association of dysregulated miR-589-3p with various diseases, such as its role in the osteogenic differentiation of periodontal ligament stem cells but not gynecological associated cancers and upregulation of this gene could be a potential novel inhibitor (68). Overall we noted that these miRNAs which are associated with differentially expressed hub genes seem to be potential diagnostic markers and therapeutic studies.

### 3.8 Network of miRNA, TF-miRNA and their target genes

**Figure 6:**
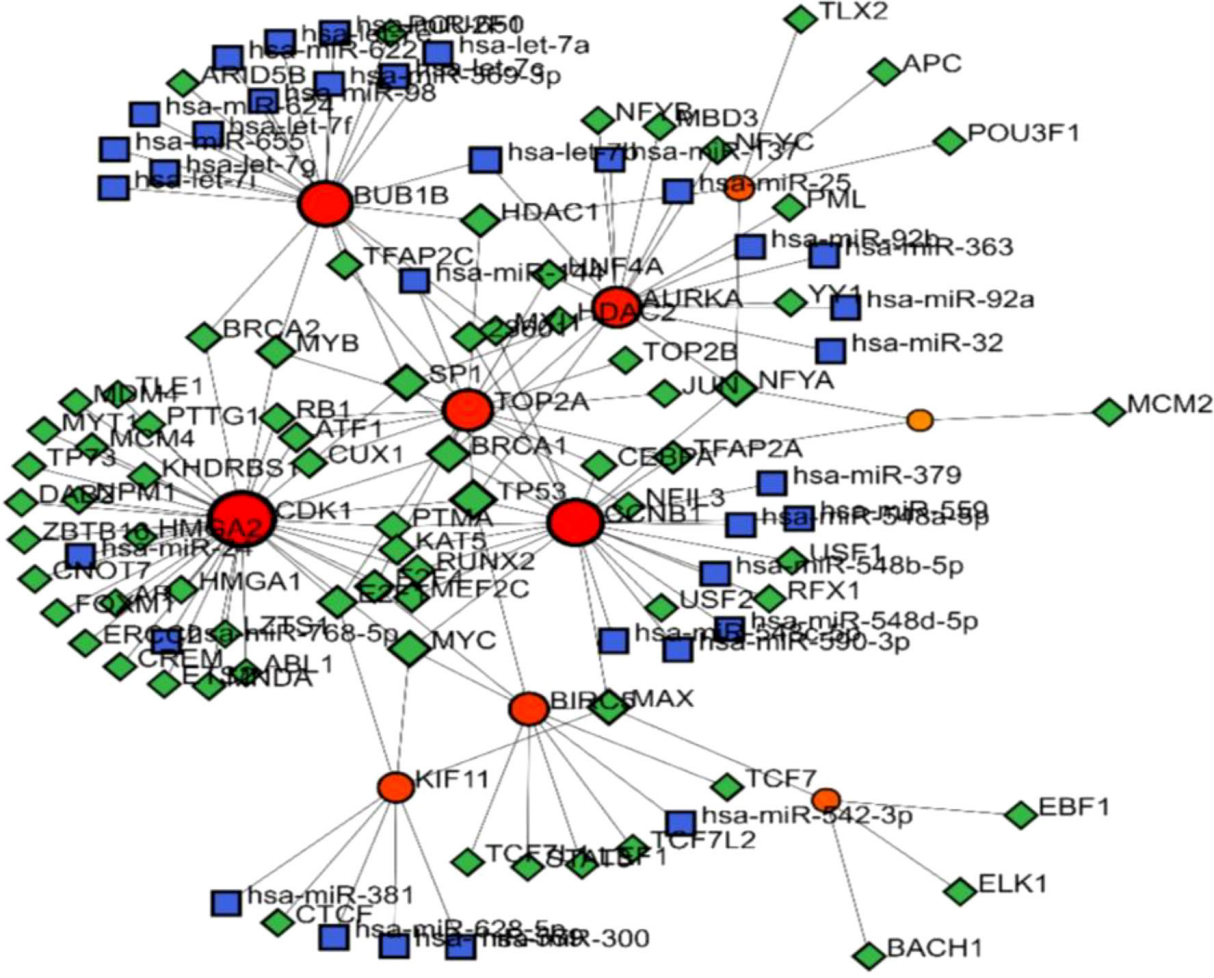
Transcription factor (TF)-miRNA coregulatory network. Complex interactions between transcription factors (shown in red circles) and microRNAs (miRNAs, shown in blue squares and green diamonds.

Network of microRNAs (miRNAs) and their target genes visualized using Cytoscape. In the network, yellow nodes represent miRNAs, while red nodes represent the target genes regulated by these miRNAs (**Figure 6**). Edges (lines) connecting the nodes indicate regulatory relationships between miRNAs and their target genes. The exact relationship between the hub gene and miRNA showed that the four genes CDK1, AURKA, CCNB1, BUB1 and BIRC5 target more miRNAs than the other hub genes, BUB1B, TOP2A, KIF11, CCNB2 and CDCAB. Among these, the highest interaction was related to BIRC5 (Grade=80), and the lowest interaction was related to BUB1B (grade=4).

Additionally, hsa-miR-653-5p, hsa-miR-495-3p,hsa-miR-381-3p, hsa-miR-1266-5p, and hsa-miR-589-3p were the top five interactive miRNAs, respectively, that targeted the most hub genes. Recent studies have highlighted that miR-653 plays a complex role in various biological processes, including cancer, by exhibiting differential expression across multiple cancer types and miR-495-3p reported to inhibit the colorectal cancer progression by HMGB1 downregulation (64, 66, 69). Similarly, a plenty of literature demonstrated the correlation between dysregulated miR-589-3p and a variety of diseases including cancer (70). This visualization helps in understanding the complex regulatory mechanisms of miRNAs that play crucial roles in gene regulation. While the hsa-miR-1266-5p is not well reported in literature but on various databases and is known to play role pericardium cancer and pericardial mesothelioma. Overall, the role of these miRNA in antioncogenic and prooncogenic functions, in various cancer types reveal that in combination of hub genes these miRNAs could be a potential targets for the diagnosis and treatment of gynelogcical cancer. While miR-495-3p which is not well studied in cancer and seems to be a novel miRNA identified in our transcriptomics data analysis and may become a potential therapeutic target for gynecological cancer following future in depth studies.

The TF-miRNA coregulatory network demonstrated that TFs and miRNAs not only regulate target genes but also influence each other, creating a dynamic and interdependent regulatory system. However, the analysis revealed that only a few TFs and miRNAs serve as major regulators, controlling disproportionately large numbers of target genes. These key regulatory hubs, such as TP53, CDK1, and AURKA, play critical roles in maintaining network functionality and stability. miRNAs are well-known for regulating cell proliferation, and alterations in these small, noncoding RNAs may facilitate tumor formation by disrupting essential cell cycle regulators (71). For example the TP53 gene, which is responsible for regulating cell cycle and cell death, is regulated by several microRNAs. Two miRNAs, miR-25 and miR-30d, directly target the 3′UTR of TP53, downregulating p53 protein levels and reducing transcriptionally activated genes. These miRNAs adversely affect apoptotic cell death, cell cycle arrest, and cellular senescence. Inhibiting these miRNAs increases p53 expression and apoptosis (72). The miR-15 family and miR-24 are notable examples of miRNAs that are frequently dysregulated in various cancers, including leukemias, hepatocellular carcinoma, prostate, colorectal, and lung cancers. Their altered expression levels can contribute to cancer progression by affecting key regulatory pathways involved in cell proliferation, apoptosis, and metastasis (73).

### 3.9 Survival analysis of upregulated miRNAs

Kaplan–Meier survival curves for patients with high and low expression levels of hsa-miR-653-5p across different cancer stages (I-IV). The p values indicate significant differences in survival probabilities between high (p = 0.0093) and low (p = 0.0018) expression groups. Kaplan–Meier survival curves for patients with high and low expression levels of hsa-miR-495-3p across different cancer stages (I-IV).

**Figure 7:**
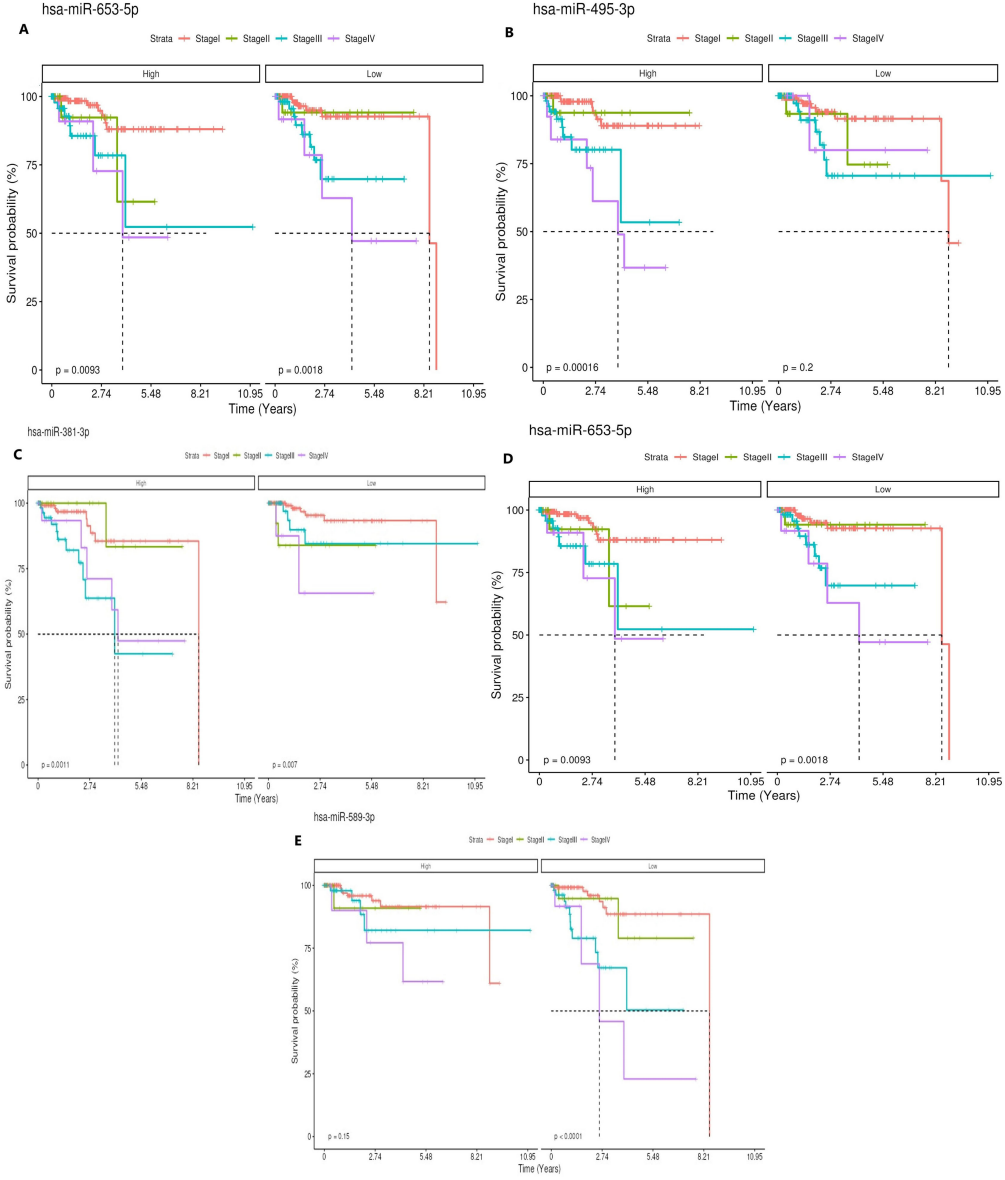
Kaplan–Meier survival analysis showing the prognostic significance of selected miRNAs in cervical cancer patients stratified by clinical stage (Stage I–IV) and expression level (high vs. low). **(A)** hsa-miR-653-5p, **(B)** hsa-miR-495-3p, **(C)** hsa-miR-381-3p, **(D)** hsa-miR-1266-5p, and **(E)** hsa-miR-589-3p.

The p values indicate significant differences in survival probabilities between high (p = 0.00016) and low (p = 0.2) expression groups. Kaplan–Meier survival curves for patients with high and low expression levels of hsa-miR-495-3p across different cancer stages (I-IV). The p values indicate significant differences in survival probabilities between high (p = 0.0011) and low (p =0.007) expression groups. Kaplan–Meier survival curves for patients with high and low expression levels of hsa-miR-495-3p across different cancer stages (I-IV). The p values indicate significant differences in survival probabilities between high (p = 0.025) and low (p =0.0011) expression groups. Kaplan–Meier survival curves for patients with high and low expression levels of hsa-miR-495-3p across different cancer stages (I-IV). The p values indicate significant differences in survival probabilities between high (p = 0.015) and low (p < 0.0001) expression groups as shown in **Figure 7**.

Many of these miRNAs circulate in extracellular vesicles and modulate cancer–associated fibroblast behavior, influencing the microenvironment such as cancer-associated fibroblasts (CAFs) are highly adaptable stromal cells that shape the tumor microenvironment by producing extracellular matrix components and secreting metabolites, growth factors, chemokines, and exosomes. Exosomal miRNAs are key mediators of this communication, influencing tumor growth, spread, therapy response, and immune evasion (74, 75). In particular, breast tumor cells release EVs containing miRNAs (e.g. miR-125b, miR-146a, miR-105) that can be taken up by nearby normal fibroblasts and reprogram them into CAFs. Conversely, CAFs themselves release EVs loaded with miRNAs (e.g. miR-500a-5p, miR-92a) that, upon uptake by cancer cells, enhance tumor proliferation, invasion, EMT, and therapy resistance (75). This bidirectional EV-miRNA exchange thus strengthens the pathological crosstalk between tumor and stromal compartments, contributing to extracellular matrix remodeling, metastatic potential, and a microenvironment conducive to tumor progression.

To confirm that indeed all four miRNAs as well as hub genes are essential for classification into good or poor prognoses, we performed the survival analysis and results showed that statistically overall survival analysis were associated with good survival rate, confirming that all four miRNAs could be potential biomarkers.

### 3.10 Validation of the hub genes

**Figure 8:**
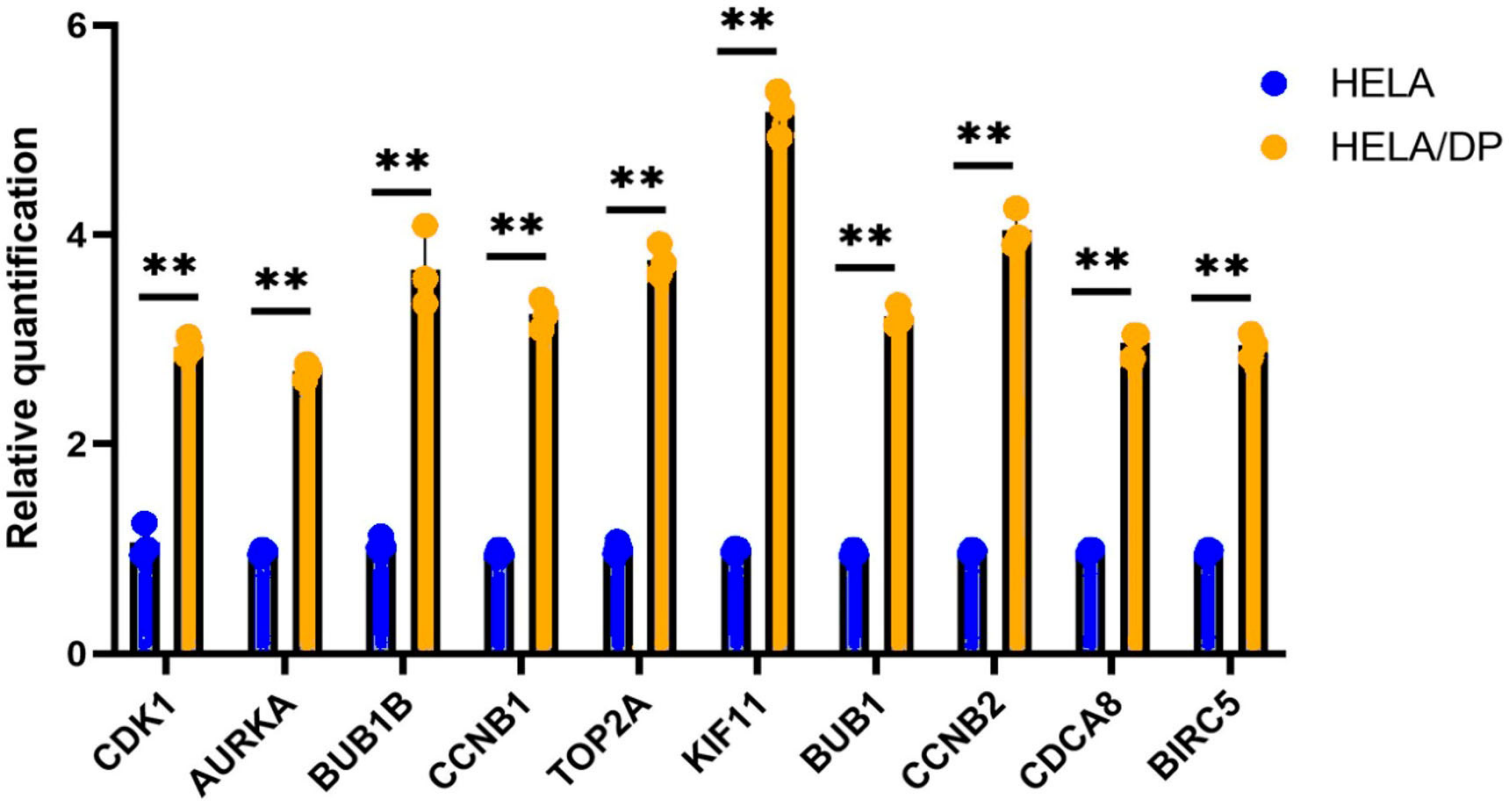
Validation of the hub genes via quantitative real time PCR analysis. **Statistical significance.

To validate the results of the bioinformatics analysis quantitative real time PCR analysis was carried out using cell lines. Compared with those in HeLa cells, the expression levels of CDK 1, AURKA, BUB1B, CCNB1, TOP2A, KIF11, BUB1, CCNB2, CDCA8 and BIRC5 in HeLa and HeLa-DPcells were significantly increased (P < 0.01) (**Figure 8**). Moreover, the raw real time PCR data such as gene expression peaks can be requested at any time. The increased expression levels of the hub genes align with the bioinformatics predictions, reinforcing the reliability of the computational analysis and highlighting the potential involvement of these genes in cancer progression and therapeutic resistance.

Although broader experimental validation such as protein level assays or functional studies would further strengthen the conclusions, the observed consistency between our bioinformatics predictions and the qRT-PCR results reinforces the reliability of the integrative analysis. As highlighted by Liu et al., that practical constraints related to time, resources, and laboratory capacity often limit the extent of immediate wet-lab validation in computational studies (76). Nevertheless, these preliminary experimental results provide important proof-of-concept support, whereas more comprehensive functional assays remain a critical and necessary direction for future translational research.

## 4 Conclusion

In gynecological cancers, dysregulation of miRNAs plays a critical role in tumorigenesis and progression. Among the upregulated miRNAs, hsa-miR-653-5p has been implicated in promoting tumor growth and metastasis in various cancers, including ovarian cancer, through its regulatory effects on key signaling pathways. Similarly, hsa-miR-495-3p is upregulated in gynecological cancer. The upregulation of hsa-miR-381-3p has been associated with tumor aggressiveness and poor prognosis in cervical cancer patients. The hsa-miR-1266-5p, hsa-miR-589-3p, and other upregulated miRNAs have been implicated in various aspects of cancer progression, including cell proliferation, invasion, and metastasis, making them promising candidates for biomarker discovery in gynecological cancers. Moreover, the increased expression levels of the hub genes aligned with the bioinformatics predictions. These dysregulated miRNAs offer promising opportunities for the development of non-invasive diagnostic tools and personalized therapeutic strategies for the management of gynecological cancers. In summary the hubs genes and miRNAs that interact with the with hub genes could have widespread effects on gene expression, highlighting their potential as key therapeutic targets. Briefly, the identified dysregulated miRNAs collectively shape key oncogenic pathways involved in proliferation, invasion, and metastasis across gynecological cancers. Integrating this miRNA hub gene axis into newer therapeutic directions offers clear opportunities: these molecules could guide biomarker-based patient stratification, inform the development of miRNA modulating therapies, and complement stromal and immune targeted strategies increasingly explored in precision oncology. In essence, the network and its broad influence on gene regulation underscores its potential as a foundation for future diagnostic and therapeutic innovations.

## Data Availability

The original contributions presented in the study are included in the article/supplementary material. Further inquiries can be directed to the corresponding authors.
